# Structural violence, decolonization and harm reduction in prisons: a critical perspective on opioid use disorders, gender and race

**DOI:** 10.3389/fsoc.2026.1819521

**Published:** 2026-06-16

**Authors:** Iván Antonio García-Montalvo, Maritza Cruz-Atayde, Diana Matías-Pérez, Marco Antonio Sánchez-Medina, Alma Dolores Pérez-Santiago, Moisés Martínez-López, Héctor Pérez-Larrañaga, Enrique López-Ramírez

**Affiliations:** División de Estudios de Posgrado e Investigación, Tecnológico Nacional de México/Instituto Tecnológico de Oaxaca, Oaxaca, Mexico

**Keywords:** decolonization, gender and race, harm reduction, opioid use disorder, prisons, structural violence

## Abstract

Substance use remains highly prevalent among prison populations. The war on drugs represents an ethical failure that has not achieved its intended goals. Redirecting resources toward reducing inequality and improving living conditions for citizens could prove more effective in addressing substance use than punitive approaches. Scientific literature consistently shows that prisoners are denied access to healthcare and face discrimination based on race, class, and gender. Continued efforts are needed to raise awareness about the benefits of opioid substitution treatment and needle exchange programs, which help reduce mortality by lowering the risk of overdose after release. Combating corruption, both inside and outside prisons, must be considered a priority. If the prevalence of drug use among incarcerated populations continues to rise, why has there been no reform of a policy long recognized as ineffective and profoundly harmful to prisoners? Who benefits from this?

## Introduction

This paper aims to critically examine the following questions: What impact do harm reduction programs implemented in prisons have on preventing overdose deaths and promoting the social reintegration of individuals with opioid use disorders? Additionally, what are the main structural and gender-related barriers that hinder their implementation and effectiveness?

The existence of gender-related barriers in prison settings is well documented. Compared to men, incarcerated women have more limited access to specific harm reduction programs and healthcare, which increases their vulnerability to mental health issues (Janati Solá et al., 2025). This situation is particularly concerning given that the female prison population has grown by over 60% since 2000. Despite this rapid increase, prison health policies continue to apply a one-size-fits-all approach designed for male bodies, ignoring biological and social differences. As a result, women’s prisons face a critical shortage of gynecological clinics, private examination rooms, and specialized personnel to ensure confidential and adequate care (Kumtanat and Rungreangkulkij, 2026). These gender-specific barriers undermine social reintegration for incarcerated women. Although understanding this barriers is essential, prior consideration must be given to the nature of substance use in prison and social reintegration.

Social reintegration is a concept distinct from just reintegration. Although the two terms are often used interchangeably in the literature, the former is broader more multidimensional: it involves providing people with opportunities on an equal footing and free from stigma. Reintegration, on the other hand, is limited to supervision and monitoring to ensure that the individual stops using substances and committing crimes.

Despite global zero-tolerance policies, the prevalence of substance use among incarcerated populations is estimated to range from 20 to 40% (Russell et al., 2022). Imprisonment is neither a useful nor a compassionate response for those living with addiction (Brown, 2022). Research has also shown, for example, that the number of adolescents in social welfare centers is associated with problematic drug use (de Tan Bibiana et al., 2020). Given the disastrous outcomes of criminalization and prohibitionist policies, it has become evident that the path of punishment and stigmatization is neither effective nor ethical (Hurley, 2019). The dominant neoliberal logic has relied on punishing individuals through incarceration via the state apparatus, ostensibly to repair harm to society and preserve order (Heiner and Tyson, 2017). If the prevalence of drug use among incarcerated populations continues to rise, why has there been no reform of a policy long recognized as ineffective and profoundly harmful to prisoners? Who benefits from this?

The persistence of punitive discourses that deny incarcerated individuals’ access to healthcare based on their criminal conduct disregards the fundamental role of systemic social and structural forces. These forces have exacerbated societal injustice and inequality, thereby disproportionately impacting marginalized groups such racialized minorities, people experiencing homeless or those with comorbid mental health disorder (Hindmarch et al., 2018). This phenomenon is understood trough the concept of structural violence as discussed in the following section. Specifically, racialized and indigenous communities have disproportionately borne the adverse consequences of systemic racism and the absence of inclusive governance, a phenomenon that fosters the development of racial resentment (Sylvestre et al., 2022). These individuals are severely punished: they are deprived of their liberty and denied access to harm reduction treatment for the crimes they committed. The authorities fail to recognize that, in contexts of extreme exclusion, being a criminal is not a choice but a result of structural violence.

These acts, often hidden from public view, represent a slow form of violence that leaves victims in a state of underdevelopment and disadvantage, ultimately leading to delayed destruction (Finley, 2021). Colonialism, gender-based violence, neoliberalism, poverty, and racism are among the structural forces that inflict harm and suffering on individuals and communities, contributing to physical and mental health problems as well as premature deaths (Moore et al., 2024). This violence destroys any possibility of social reintegration or a productive future, leading instead to a slow death through neglect and exclusion. Yet the system continues trying to force them to stop using drugs without offering anything in return.

This leads us to analyze incarceration and access to harm reduction programs through the lens of drug decriminalization. If criminalizing drugs has already been proven an ethical failure (Hauskeller and Sjöstedt-Hughes, 2022), then it is essential to broaden our perspective, conduct more qualitative research, and raise awareness about the human benefits prisoners’ experience when they receive harm reduction treatment. These benefits have often been overshadowed by policies that prioritize capital, industry, and bureaucracy over what should be the government’s central goal: the wellbeing of individuals and the community (Hauskeller and Sjöstedt-Hughes, 2022).

Although release from prison continues to pose a high risk of overdose, there is sufficient evidence that Opioid Substitution Treatment (OST) can help mitigate the danger, especially for those who have already received treatment during their incarceration (Geißelsöder et al., 2024). However, the effect on overdose mortality does not persist in the long term due to various risk factors, including serious mental illness or having been discharged from high-security units (Bukten and Stavseth, 2024). The implementation of punitive drug policies contributes to a disproportionate concentration of individuals with or at high risk for HIV in correctional facilities, a phenomenon linked to the prevalence of injection drug use and associated risk behaviors (Rozanova et al., 2018). Prohibition has been unable to prevent drug use not only in the general community but also in prison settings. High levels of injecting drug use continue to be recorded in prisons in all regions of the world (Sander et al., 2019). Prisoners are criminalized, but corruption is not addressed when it comes to prison staff who allow drugs to enter or be sold inside. It is the poor and the marginalized who are treated as criminals and punished, even though crime also operates from the highest levels.

## Structural violence

Structural violence encompasses whatever prevents individuals from developing their capacities, dispositions, or possibilities (Winter, 2012). Prisons worldwide confine people who face multiple disadvantages-health issues, poverty, mental illness, unemployment, lack of social support, and limited access to medical care (Fraser, 2011)—and these are also spaces marked by racial and gender violence (Pellow, 2021).

For example, incarcerated indigenous women constitute a health-underserved population, leading to a worse standard of living than other prison populations (Sullivan et al., 2019). Dying from a treatable disease without access to medicines, or a group having a lower than average life expectancy due to poverty or inequality, is also structural violence (Sylvestre and Castleden, 2022). Similarly, heroin users in prison face greater structural vulnerability due to social inequalities related to race, class, poverty, and the criminalization of drug use, which contribute to biopsychosocial deterioration (Romero-Mendoza et al., 2022), all while the medical-industrial complex takes advantage of the overdose crisis as a market opportunity (Kavanaugh, 2022).

## Decolonizing prison?

Abolitionist theory emerges from social movements that have long worked toward ending incarceration and transforming violent practices directed at drug users (Singh Kelsall et al., 2024). It recognizes that drug prohibition-the “War on Drugs”—is widely viewed not only as a counterproductive public health measure but also as a mechanism of systemic oppression (Dertadian, 2024). Consequently, a methodological and cultural shift in drug policy is urgently needed to counteract the racist, imperialist, and socioeconomic logic that currently shapes drug policy in North America (Singh Kelsall et al., 2024). This reality is deeply intertwined with the coloniality of power—the persistence and reproduction of colonial-era domination within contemporary society—a phenomenon particularly evident in institutional contexts such as policing and the criminal legal system (Cunneen, 2023).

Decolonization can be understood as a term that encompasses various efforts to resist the distinct but intertwined processes of colonization and rationalization, to bring about transformation and reparation in reference to the history and culture of humanity (Stein and Andreotti, 2016). It focuses on examining concepts of power and access to opportunities, while critically questioning the systems and structures that have perpetuated inequalities caused by internalized oppression, known as the “wound of the soul” (Singh et al., 2020). In this sense, the decolonization of drug policies involves dismantling colonial power structures and replacing them with strategies based on science, health, education, and social equity (Lasco, 2022). This aligns with the idea that abolition occurs when the established order is broken and, at the same time, one is faced with undeveloped land (Heiner and Tyson, 2017)—an abolition that may consist of incarceration combined with support networks, implying a total transformation, investment in housing, work, health, and, in general, conditions that reduce social disadvantage and marginalization (de Rege, 2024).

## Harm reduction programs in prisons

Between 2001 and 2018, overdose-related deaths in U.S. prisons increased by 600% (Nall et al., 2024). An estimated 65% of incarcerated individuals meet the criteria for substance use disorder, and 20% are imprisoned specifically for drug-related offenses (Wogen and Restrepo, 2020). Upon incarceration, many individuals are abruptly cut off from their substance of use, resulting in untreated withdrawal symptoms when pharmacological interventions—such as methadone, buprenorphine, or naltrexone—are not provided (Marotta et al., 2023).

The harm reduction model seeks to mitigate the negative consequences of drug use by acknowledging that people who use drugs are part of society. For instance, instead of punishing someone for being found in possession of using drugs, harm reduction programs provide clean syringes to prevent the transmission of HIV. Its goal is to ensure their safety and wellbeing rather than to ignore, condemn, or criminalize them (Gibson and Emmert, 2022). Preventive interventions for sexually transmitted and blood-borne infections benefit not only incarcerated populations but also the broader community (Golrokhi et al., 2018).

Evidence indicates that methadone treatment improves the social wellbeing of incarcerated individuals and their families, while also reducing drug-related crime, violence, self-harm, and expenditure on illicit substances (SaberiZafarghandi et al., 2024). In both community and prison settings, it is recommended that people who inject drugs (PWID) receive annual screening for infectious diseases (Stöver et al., 2021). Harm reduction measures include the provision of condoms, disinfectants, and opioid substitution therapies such as methadone or buprenorphine (Lines et al., 2005).

Prison Needle Exchange Programs (PNEPs) are harm reduction initiatives designed to prevent needle sharing. Despite over 20 years of implementation, they remain highly controversial (Treloar et al., 2016). Nevertheless, these programs can contribute to making prisons more humane and less stressful environments by providing treatment for substance use disorders (Armstrong-Mensah et al., 2021). They have become a primary intervention for chronic opioid dependence in prisons, particularly in the Netherlands, Australia, Spain, and Canada (Zaller et al., 2022).

Over the past three decades, research has consistently shown that needle exchange programs in prisons do not lead to an increase in injecting drug use. Moreover, there have been no documented cases of needles being used as weapons against staff or other inmates (Michaud and van der Meulen, 2023). Despite strong evidence supporting the effectiveness of Opioid Agonist Therapy (OAT) and prison needle and syringe programs (Rance et al., 2021), these interventions continue to face resistance and lack of acceptance in carceral settings (Giffin et al., 2023). The barriers mentioned above are particularly acute for women, who fase gender-specific obstacles to harm reduction treatment. Overdose among women has increased by more than 500% since the 1990s, and in recent years women have accounted for 31.4% of overdose deaths (Skogseth et al., 2024).

Women with opioid use disorder face additional structural barriers to accessing MOUD beyond those typically encountered in prisons, including high rates of trauma and sexual exploitation, as well as severe stigma related to motherhood and the loss of child custody (Fiddian-Green et al., 2022). According to the most frequently reported barriers, Black and Hispanic women face significant disadvantages compared to white women (Scheidell et al., 2024).

There is broad clinical consensus that Opioid Maintenance Treatment (OMT) is highly effective in reducing opioid use, curbing the transmission of infectious diseases, and enhancing social functioning (Soyka and Laber, 2025). This approach has received endorsements from the World Health Organization, the National Institute on Drug Abuse, and the National Academy of Medicine (Berk et al., 2025). Legal recognition of its importance is evident in a ruling by the European Court of Human Rights, which determined that denying drug treatment to individuals constitutes inhuman treatment (Stemmler et al., 2024). Empirical studies further support this, showing that individuals who received opioid medication during treatment had significantly lower rates of illicit opioid use between 3 months and 1 year after release. Nevertheless, structural barriers such as unemployment and recidivism persist (Stemmler et al., 2024). In addition to their effectiveness in reducing infections and preventing overdoses, the benefits of these harm reduction interventions extend beyond prison settings.

## After prison

The World Health Organization, the United Nations Office on Drugs and Crime (UNODC), and UNAIDS recommend a comprehensive approach to harm reduction that includes: needle and syringe programs; opioid substitution therapy; HIV testing and counseling; antiretroviral therapy; prevention and treatment of sexually transmitted infections; condom distribution for people who inject drugs and their sexual partners; information, education, and communication initiatives for this population; diagnosis, treatment, and vaccination for viral hepatitis; and prevention, diagnosis, and treatment of tuberculosis (Joint United Nations Programme on HIV/AIDS (UNAIDS), 2019). However, despite these international guidelines, it has been reported that some institutions prohibit social organizations from distributing informational brochures or other educational materials that describe safe injection practices (der Van Meulen et al., 2016).

The risk of death from opioid overdose increases more than sevenfold in the first 2 weeks following release from prison (Rozanova et al., 2018; Cepeda et al., 2020). Individuals with opioid use disorder who are released from incarceration face an elevated risk of overdose due to the loss of physical tolerance resulting from interrupted opioid use during their imprisonment (Grella et al., 2021). It is therefore critically important that people with opioid use disorder in the criminal justice system receive effective treatment prior to release (Moore et al., 2019). To reduce this risk, programs such as Take-Home Naloxone provide kits and training to families, enabling them to assist their relatives in the event of an opioid overdose after release (Grella et al., 2021).

Despite the existence of international recommendations, practices differ widely across countries. For example, Mexico became the first country in Latin America to adopt an HCV elimination strategy. That same year, Mexico purchase 12,500 direct-acting antiviral treatments for an estimated population of 550,000 infected people (Marquez et al., 2021). However, that same year, the federal government announced that it was suspending funding for civil society organizations responsible for providing HIV treatment and prevention services (Cepeda et al., 2020). Meanwhile, recent changes in public policy in countries such as Canada now allow transgender or non-binary individuals to be admitted to federal prisons according to their gender expression, rather than their anatomy (Paynter et al., 2022). These examples illustrate that, despite the existence of international guidelines, the absence of governmental commitment to resource allocation for harm reduction programs ensures that progress will remain exceedingly slow given the magnitude of the opioid use crisis in prisons—a situation that carries a devastating human toll.

## Conclusion

In this article, we have argued that punitive policies and prison systems have failed to adapt their infrastructure to meet the specific needs of women, and that systemic racism widens the gap, thereby hindering equitable access to harm reduction programs for the entire population. This situation calls for a fundamental break with the long-standing tradition of responding to crime through harsh and often dehumanizing punishment. As with many complex social issues, academics and researchers have a crucial role to play: not only in producing knowledge, but in translating their findings into concrete proposals that can help transform a system that has too often abused its power, dominating and humiliating those who belong to marginalized communities. These include decriminalizing drug possession, expanding harm reduction programs even after release.

Opioids are prescribed to treat physical pain, yet many people who develop opioid use disorder use them to numb deeper wounds—those produced by structural violence that affects entire families, with children often bearing the heaviest burden. The state also has a moral responsibility to provide equitable access to evidence-based treatment options within prisons, ensuring that care is offered on equal terms, grounded in respect for human dignity. In this sense, the cycle of addiction, overdose, and death constitutes a form of structural violence resulting from the failure of public policies.
